# Introduction to Editorial Board Members: Dr. Raghunath Mashelkar

**DOI:** 10.1002/btm2.10081

**Published:** 2017-10-17

**Authors:** Samir Mitragotri

**Affiliations:** ^1^ Harvard University Cambridge, MA 02138

In this issue of *Bioengineering & Translational Medicine*, we proudly feature Dr. Raghunath Mashelkar. He is the President of Global Research Alliance, world's largest network of Research and Technology Organizations dedicated to creating global good through global knowledge partnerships. He is also the Chairperson of the National Innovation Foundation of India, which promotes grassroots innovation, implying innovations by the people done for the people.

Dr. Mashelkar is known globally for his pioneering research in polymer science and engineering, for his exceptional and transformative leadership of major Indian R&D institutions, his playing a key role in pioneering the intellectual property movement in India, and for his being a dominant force in shaping India's Science and Technology policies and performance in the post‐liberalized India.

Dr. Mashelkar launched the first Indian school of polymer science and engineering research in early 80s, which is now internationally recognized. It fully integrated polymer chemistry and physics, polymer reaction engineering, polymer processing, rheology, non‐Newtonian fluid mechanics, modeling, and simulation into a single research program. Dr. Mashelkar has himself made path‐breaking contributions in transport phenomena and thermodynamics of swelling, superswelling, and shrinking polymers, modelling of polymerization reactors, and engineering analysis of non‐Newtonian flows.

Dr. Mashelkar has been honored by several recognitions including election as Fellow of Royal Society, Foreign Associate of US National Academy of Science as well as US National Academy of Engineering, Foreign Fellow of Royal Academy of Engineering (UK), Australian Academy of Technological Science and Engineering and American Academy of Arts and Science. Besides becoming the President of Indian National Science Academy, 37 universities have honored Dr. Mashelkar with DSc (Hon. Causa), which include Universities of London, Wisconsin, Pretoria, Monash, and Swinburne, among others.

As Director of India's National Chemical Laboratory (NCL) (1989–1995), Dr. Mashelkar gave a new orientation to NCL's research by shifting the emphasis from import substitution to creating globally competitive technologies and international patenting. Under his leadership, NCL became the first laboratory from India to do the reverse transfer of technology by licensing its patents to leading U.S. companies for technologies within their core expertise.

As Director General of Council of Scientific and Industrial Research, CSIR (1995–2006), Dr. Mashelkar conceived and successfully led the process of transformation of CSIR. He masterminded the white paper “CSIR 2001: Vision & Strategy” setting up a game changing agenda. The story of the transformation of CSIR (to a user focused and performance driven organization that worked as a ‘Team CSIR’ rather than as a set of isolated individual laboratories) was recognized by the Indian industry through the J.R.D. Tata Corporate Leadership Award (1998) to Dr. Mashelkar and also by the global industry, when Dr. Mashelkar received the Star of Asia Award of Business Week (United States) at the hands of President George Bush (Sr) in 2005.

Dr. Mashelkar created an awakening of Intellectual Property Rights in Indian research laboratories, academia and industry. The patenting culture propagated by him spread deep and wide. From an insignificant player in the intellectual property space, CSIR held the distinction of being the holder of around 60–70% of the U.S. patents granted to India during Dr. Mashelkar's tenure.

Dr. Mashelkar played a leading role in formulating the national intellectual property rights policies. He led the successful revocation of the U.S. patent on wound healing properties of *turmeric* and also the revocation of the U.S. patent on Basmati rice. He also masterminded the creation of India's Traditional Knowledge Digital Library, a unique first.

Dr. Mashelkar is a career‐long crusader for innovation and innovation‐led growth and development. He is India's most active and vocal proponent of science led innovation. Powered by extreme optimism and infectious enthusiasm, he is on a mission to educate the national audience, from classrooms to boardrooms, about the power of innovation. Through countless public lectures, authoritative books, television interviews, and engagement in social media, he has actively encouraged the young generation of researchers to follow the path of innovation. At the same time, he has also actively promoted ”inclusive innovation,” which aims to achieve more from less for more people, not just for more profit. Last year, Dr. Mashelkar was elected as a fellow of the US National Academy of Inventors (NAI). He is the first person from India to be elected as a fellow of NAI.

While Dr. Mashelkar's scientific accomplishments are remarkable by any measure, his service to the nation is truly pathbreaking! Dr. Mashelkar has chaired 12 committees established by the Indian Government to seek guidance on various matters of national interest ranging from auto fuel policies, to drug regulation policies, to sanitation technologies. Dr. Mashelkar chaired the technical inquiry committees set up to investigate some of world's worst industrial disasters, the Bhopal gas tragedy (1984) where thousands died overnight and the MGCC explosion (1992), where 34 people lost lives.

Dr. Mashelkar has been a member of the Science Advisory Council to the Indian Prime Minister. His impact on scientific issues of national significance has been recognized by the Government of India by several honors including the Padma Vibhushan, the Padma Bhushan, and the Padma Shri. The first of these awards is the second highest civilian honor bestowed by the Indian Government to an individual.

One of Dr. Mashelkar's major contributions to science is his transformative impact on public view of science and technology in India. With his extreme passion for innovation, inspiring speaking skills, and deep understanding of societal needs, he has become an ambassador for science and technology to the society.

January 1, 2018 marks the 75th birthday of Dr. Mashelkar. We take this opportunity to send our warm wishes to this visionary innovation activist of India and thank him for his long‐standing dedication to the cause of advancing science and technology through multiple avenues including pioneering research in polymer science and engineering, leading educational and research institutions in India, raising awareness about innovation among various stakeholders, and inspiring a generation of young researchers to engage in science and technology. We thank him for his participation in *Bioengineering & Translational Medicine* Editorial Advisory Board and look forward to his continued support in future.

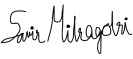

**Figure 1:**
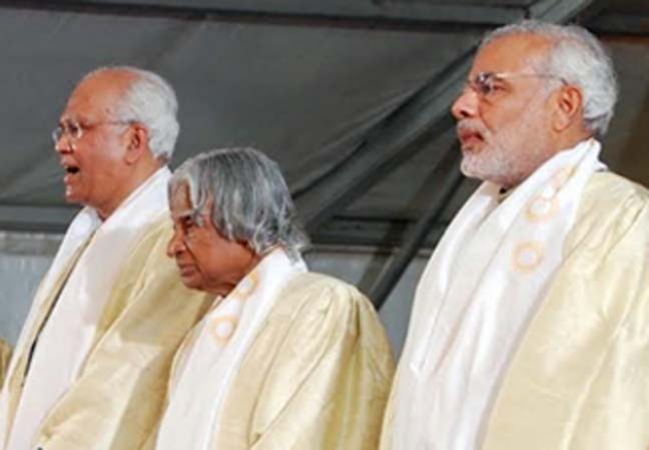
Dr. Mashelkar on stage at an event with former Indian President Dr. Abdul Kalam and current Indian Prime Minister Narendra Modi

**Figure 2:**
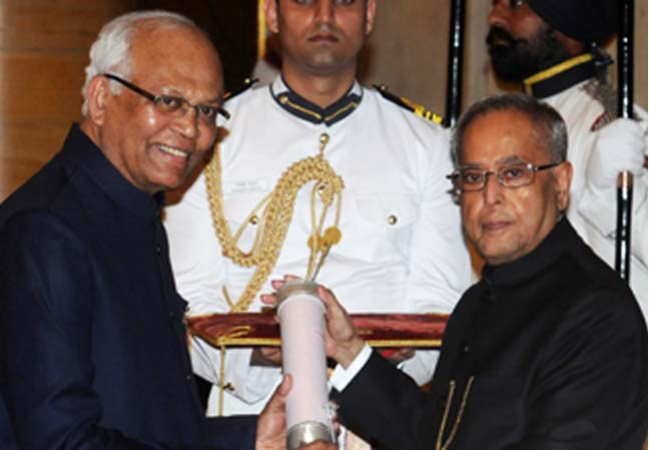
Dr. Mashelkar receiving Padma Vibhushan award from former Indian President Pranab Mukherjee

